# Risk Factors for Postoperative Hyphema Following Baerveldt Glaucoma Implant Surgery: A Retrospective Cohort Study

**DOI:** 10.3390/jcm15062247

**Published:** 2026-03-16

**Authors:** Kentaro Iwasaki, Ayami Katsuo, Shogo Arimura, Yoshihiro Takamura, Masaru Inatani

**Affiliations:** Department of Ophthalmology, Faculty of Medical Sciences, University of Fukui, Fukui 910-1193, Japan; kenkentaro0329@yahoo.co.jp (K.I.); a-tomoda@g.u-fukui.ac.jp (A.K.); leosunshine33@gmail.com (S.A.); ytakamura@hotmail.com (Y.T.)

**Keywords:** baerveldt glaucoma implant, hyphema, tube insertion site, intraocular pressure, glaucoma surgery, risk factors

## Abstract

**Background/Objectives**: To investigate the incidence and risk factors for postoperative hyphema following Baerveldt glaucoma implant (BGI) surgery. **Methods**: This retrospective study included Japanese patients who underwent BGI surgery at Fukui University Hospital between 1 April 2012 and 31 March 2025. Hyphema was defined as any clinically detectable blood in the anterior chamber. Baseline demographic, ocular, and surgical variables were compared between eyes with and without hyphema. Independent risk factors for hyphema were determined using multivariable logistic regression analysis. **Results**: Of 273 eyes, 77 (28.2%) developed postoperative hyphema. On multivariable analysis, tube insertion site and intraocular pressure (IOP) on postoperative day 1 were identified as independent predictors. Although the overall effect of tube insertion site was borderline (*p* = 0.074), anterior chamber (odds ratio [OR], 2.83; *p* = 0.036) and ciliary sulcus insertion (OR, 2.88; *p* = 0.031) were associated with significantly higher risk of hyphema compared with vitreous cavity insertion. Lower postoperative day 1 IOP was also a significant predictor (*p* < 0.01). Patient-related factors, including age, diabetes mellitus, hypertension, antithrombotic therapy, neovascular glaucoma (NVG), combined surgery, number of previous intraocular surgeries, and preoperative IOP, were not independently associated with hyphema. In a sensitivity analysis excluding NVG eyes (n = 191), vitreous cavity insertion remained protective, and postoperative day 1 IOP remained an independent predictor. **Conclusions**: Postoperative hyphema following BGI surgery is primarily determined by surgical factors and early postoperative IOP. Vitreous cavity tube insertion is associated with a markedly lower hyphema risk than anterior chamber or ciliary sulcus insertion.

## 1. Introduction

Glaucoma drainage devices are increasingly used for the surgical management of refractory glaucoma worldwide [1,2,3], particularly in cases with previous ocular surgery or high risk of filtering failure. Among these devices, the Baerveldt glaucoma implant (BGI) is widely used because of its favorable long-term intraocular pressure (IOP) control [4]. However, postoperative complications remain a concern, particularly during the early postoperative period.

Hyphema is a common complication after tube shunt surgery, with most cases occurring in the early postoperative period and resolving spontaneously. The reported frequency of hyphema after tube shunt surgery ranges from approximately 2% to 39% [5,6,7,8,9,10,11,12,13]. Although most cases are transient, hyphema can cause visual disturbance, IOP instability, and patient discomfort in the early postoperative period, potentially affecting activities of daily living, particularly in patients with impaired vision in the fellow eye.

In trabeculectomy, hyphema has been extensively investigated, and several studies have identified preoperative risk factors, including neovascular glaucoma (NVG), exfoliation glaucoma, high preoperative IOP, anticoagulant or antiplatelet medication use, and anterior chamber neovascularization [14,15,16,17]. In the eyes with NVG, intravitreal administration of anti-vascular endothelial growth factor agents has been reported to reduce the incidence of postoperative hyphema by inducing regression of anterior segment neovascularization [14,18,19]. These findings have contributed to improved perioperative management and risk stratification in patients undergoing trabeculectomy.

Glaucoma drainage device surgery, such as BGI placement, differs fundamentally from trabeculectomy in both surgical technique and aqueous outflow pathway. Unlike trabeculectomy, BGI surgery does not require excision of the trabecular meshwork or routine peripheral iridectomy, although peripheral iridectomy may occasionally be performed in selected cases, such as when the tube is inserted into the ciliary sulcus in pseudophakic eyes to prevent tube occlusion by the iris. Instead, a silicone tube is inserted into the eye, and aqueous humor is diverted through the tube toward a posteriorly positioned endplate, where filtration occurs. The tube insertion itself may introduce unique sources of postoperative bleeding depending on the insertion site, including the anterior chamber, ciliary sulcus, or vitreous cavity. In addition, systemic factors such as hypertension and the use of anticoagulant therapy may influence the risk of postoperative hyphema in eyes undergoing BGI surgery. However, despite the increasing use of BGI for refractory glaucoma, the incidence and risk factors for hyphema following BGI surgery have not been systematically evaluated.

To date, no studies have specifically analyzed preoperative and intraoperative risk factors for hyphema after BGI surgery using multivariable statistical models. Therefore, this study aimed to investigate the clinical factors associated with postoperative hyphema following BGI surgery, with particular attention to patient characteristics, glaucoma subtype, systemic comorbidities, anticoagulant use, and tube insertion site.

## 2. Materials and Methods

### 2.1. Patient Selection

This retrospective cohort study was reviewed and approved by the Institutional Review Board of Fukui University Hospital and conducted in accordance with the principles of the Declaration of Helsinki. Owing to its retrospective design, the requirement for informed consent for participation in this study was waived.

We retrospectively reviewed patients who underwent BGI surgery with a 350-mm^2^ endplate (BG101-350 or BG102-350; Abbott Medical Optics, Abbott Park, IL, USA) at Fukui University Hospital between 1 April 2012, and 31 March 2025. Eligible participants were adults aged 20 years or older. Eyes with no light perception, a history of previous tube-shunt surgery (including BGI or Ahmed glaucoma valve), or insufficient postoperative follow-up (<1 month) were excluded to ensure adequate assessment of early postoperative complications, including hyphema occurring within the first postoperative week. When both eyes of a patient met the eligibility criteria, only the eye that underwent surgery first was included to ensure statistical independence.

### 2.2. Surgical Procedure

The surgical procedure has been described previously [10]. All tube shunt procedures were performed using BG101-350 or BG102-350 end plates. The silicone tube was ligated using an 8-0 absorbable vicryl suture to prevent early postoperative hypotony. A fornix-based conjunctival flap was created after subconjunctival and sub-Tenon’s anesthesia with xylocaine. The implant plate was positioned in the superotemporal quadrant and secured to the sclera approximately 10 mm posterior to the corneal limbus, and placed under the rectus muscles. A scleral tunnel was created using a 23-gauge needle, and the tube was inserted into the anterior chamber or ciliary sulcus. In eyes with a history of vitrectomy, the tube was inserted into the vitreous cavity through the pars plana. For pars plana tube insertion, the tube with the Hoffmann elbow (BG102-350) or straight tube (BG101-350) was inserted into the vitreous space using a 20- or 24-gauge microvitreoretinal-lance. Sherwood slits were created in the tube using a 9-0 nylon needle to reduce the incidence of early postoperative IOP elevation. The tube was covered with a half-thickness rectangular self-scleral flap or a preserved scleral patch graft obtained from the eye bank. The scleral patch and conjunctival flap were sutured using 9-0 nylon and 8-0 absorbable vicryl sutures.

### 2.3. Data Collection

We collected patient demographic, ocular, and surgical data, including sex, age, glaucoma subtype, lens status, tube insertion site, plate type and position, preoperative and postoperative IOP, number of glaucoma medications, history of previous intraocular surgery, and combined surgery status. Systemic comorbidities, including hypertension and diabetes mellitus, as well as antithrombotic therapy (including anticoagulant and antiplatelet medications), were also recorded. Postoperative examinations were performed daily during hospitalization for the first postoperative week and subsequently at regular outpatient visits. Therefore, hyphema was assessed during this early postoperative period. Because of the retrospective design of this study, the examiners were not masked to the patients’ clinical characteristics or surgical factors.

### 2.4. Outcome Measures

The main aim was to investigate risk factors for hyphema as an early postoperative complication (within 1 week) after BGI surgery. Postoperative hyphema was defined as any clinically detectable blood in the anterior chamber observed on slit-lamp examination.

### 2.5. Statistical Analysis

Categorical variables were analyzed using the chi-squared test or Fisher’s exact test, and continuous variables were compared using the Mann–Whitney U test. Multivariable logistic regression analysis was performed to identify factors associated with postoperative hyphema. A *p*-value < 0.05 was considered statistically significant. All analyses were performed using JMP Pro software (version 17.2.0; SAS Institute Inc., Cary, NC, USA).

## 3. Results

### 3.1. Patient Characteristics

A total of 273 eyes from Japanese patients were included in the analysis, and postoperative hyphema developed in 77 eyes (28.2%). Most cases were transient and resolved spontaneously; however, anterior chamber irrigation was required in two eyes at postoperative week 1. Baseline patient characteristics and comparisons between eyes with and without hyphema are summarized in Table 1. Eyes with postoperative hyphema had significantly lower IOP on postoperative day 1 compared with those without hyphema (16.4 ± 13.0 vs. 22.6 ± 12.5 mmHg; *p* < 0.01). No other significant differences were observed between the two groups.

### 3.2. Risk Factor Analysis

Patient characteristics, including age, antithrombotic therapy, hypertension, diabetes mellitus, glaucoma type, tube insertion site, combined surgery, preoperative IOP, postoperative day 1 IOP, and number of previous intraocular surgeries, were assessed as potential determinants of postoperative hyphema. On multivariable logistic regression analysis, tube insertion site and IOP on postoperative day 1 were independently associated with postoperative hyphema (Table 2). Compared with vitreous cavity insertion, anterior chamber insertion (odds ratio [OR], 2.83; 95% confidence interval [CI], 1.07–7.86; *p* = 0.036) and ciliary sulcus insertion (OR, 2.88; 95% CI, 1.10–7.82; *p* = 0.031) were associated with significantly higher risk of hyphema. Lower IOP on postoperative day 1 was also significantly associated with hyphema development (OR, 0.96; 95% CI, 0.93–0.98; *p* < 0.01). Although the overall effect of tube insertion site showed borderline significance (*p* = 0.074), pairwise comparisons demonstrated significantly increased risk of hyphema with anterior chamber and ciliary sulcus insertion relative to vitreous cavity insertion. No other variables were independently associated with postoperative hyphema.

### 3.3. Sensitivity Analysis Excluding Neovascular Glaucoma

Because NVG represents a distinct pathological condition characterized by anterior segment neovascularization that may independently predispose to postoperative bleeding, a sensitivity analysis excluding eyes with NVG (n = 191) was performed to evaluate the robustness of the primary findings (Table 3). Tube insertion site and IOP on postoperative day 1 remained independently associated with postoperative hyphema. Compared with vitreous cavity insertion, anterior chamber insertion (OR, 16.16; 95% CI, 2.59–321.79; *p* < 0.01) and ciliary sulcus insertion (OR, 22.75; 95% CI, 3.43–465.01; *p* < 0.01) were associated with markedly higher risk of hyphema. Lower IOP on postoperative day 1 also remained a significant predictor of hyphema (OR, 0.96; 95% CI, 0.93–0.99; *p* < 0.01). No other statistically significant determinants were identified in this analysis. These findings confirm that the associations between tube insertion site, early postoperative IOP, and hyphema were not driven by NVG.

## 4. Discussion

In this retrospective cohort study, we investigated clinical factors associated with postoperative hyphema following BGI surgery. To our knowledge, this is the first study to systematically evaluate preoperative and intraoperative risk factors for hyphema following BGI surgery using multivariable statistical models. Our results demonstrated that the tube insertion site and IOP on postoperative day 1 were independently associated with hyphema development. Specifically, anterior chamber and ciliary sulcus tube insertion were associated with significantly higher risk of hyphema compared with vitreous cavity insertion, and lower early postoperative IOP was a significant predictor. In the full cohort, the overall effect of tube insertion site showed borderline statistical significance. However, after excluding eyes with NVG, the association became more pronounced. This suggests that heterogeneous bleeding mechanisms in NVG eyes may partially obscure the effect of tube insertion site in the overall analysis. In the sensitivity analysis excluding NVG eyes, anterior chamber and ciliary sulcus tube insertion remained significantly associated with hyphema development, supporting the robustness of the primary findings.

The present findings suggest that vitreous cavity tube placement is associated with a substantially lower risk of postoperative hyphema. A previous study comparing pars plana and anterior chamber placement of glaucoma drainage devices has also reported a lower tendency for postoperative hyphema with pars plana tube insertion [20]. One possible explanation is that vitreous insertion avoids direct contact between the tube and highly vascularized anterior segment tissues, such as the iris and ciliary body, thereby reducing mechanical irritation and the likelihood of bleeding. In contrast, anterior chamber and ciliary sulcus insertion may increase the risk of microtrauma to adjacent vascular structures, particularly in eyes with fragile iris vasculature or postoperative inflammation. In addition, posterior tube placement may be less influenced by conjunctival or anterior segment inflammation, which could further contribute to its protective effect. Another possible explanation is that vitreous cavity tube insertion is often performed in eyes that have undergone pars plana vitrectomy or combined vitrectomy procedures. In such eyes, the altered intraocular environment after vitrectomy may influence postoperative inflammatory or hemorrhagic responses, which could potentially reduce the likelihood of anterior chamber bleeding.

Lower IOP on postoperative day 1 was also independently associated with hyphema. Early postoperative hypotony may promote choroidal expansion or alter intraocular pressure gradients, potentially predisposing fragile intraocular vessels to rupture [21]. This finding highlights the importance of careful early postoperative IOP management following BGI surgery.

Notably, patient-related factors, including age, diabetes mellitus, hypertension, antithrombotic therapy, and NVG, were not independently associated with postoperative hyphema in multivariable analysis. These results suggest that surgical and early postoperative factors may play a more critical role than baseline systemic or ocular characteristics in determining hyphema risk after BGI placement. Although NVG is generally considered a strong risk factor for postoperative hyphema after filtering surgery [14], it was not independently associated with hyphema in this study. This may be partly explained by the fact that intravitreal anti–vascular endothelial growth factor agents were administered within 1 month before surgery in half of NVG eyes (41 of 82 eyes), which may have suppressed anterior segment neovascularization. In addition, vitreous cavity tube insertion was performed in a substantial proportion of NVG eyes (64.6%), which may also have reduced the likelihood of anterior chamber bleeding. Consequently, the incidence of hyphema in NVG eyes may have been comparable to that in other glaucoma subtypes. Similarly, systemic factors such as hypertension, diabetes mellitus, and antithrombotic therapy were not significant predictors. Although antithrombotic therapy has been reported to increase postoperative hemorrhagic complications after tube shunt surgery [22], it was not independently associated with hyphema in our multivariable model. This may be because local surgical factors, particularly tube insertion site and early postoperative IOP, exerted a stronger influence on hyphema development than systemic bleeding risk in eyes undergoing BGI surgery. In our cohort, antithrombotic therapy was generally not discontinued perioperatively, which may further support the notion that systemic bleeding tendency played a limited role in postoperative hyphema in this surgical setting.

This study has several limitations related to its retrospective design. First, we did not quantify the volume or duration of postoperative hyphema, nor did we differentiate between subtle and extensive hyphema in the anterior chamber. Second, postoperative management, including medical treatment during the early postoperative period, was not standardized and was left to the discretion of the treating surgeon. Therefore, unmeasured differences in postoperative care may have influenced hyphema development. In addition, detailed information regarding the type, dosage, and duration of antithrombotic therapy was not consistently available, and differences among specific antiplatelet or anticoagulant agents could not be evaluated. Similarly, the indication and timing of preoperative anti-vascular endothelial growth factor therapy in NVG eyes were not standardized, which may have influenced the observed association between NVG and postoperative hyphema. Third, in the sensitivity analysis excluding NVG eyes, the confidence intervals for the effect of tube insertion site were relatively wide. This likely reflects the limited number of events in the vitreous cavity insertion group, resulting in reduced statistical precision. Therefore, the magnitude of this association should be interpreted with caution. Fourth, surgeries were performed by experienced glaucoma surgeons at our institution. Although the fundamental surgical techniques were standardized, minor procedural variations between surgeons or changes in surgical practice over the long study period may have occurred and could have influenced postoperative complication rates. Finally, this was a single-center study conducted in a Japanese population, which may limit the generalizability of our findings. Further multicenter prospective studies are required to address these limitations.

From a clinical perspective, the present findings may have implications for surgical planning and perioperative management in BGI surgery. Our results suggest that surgical factors, particularly tube insertion site and early postoperative IOP control, play an important role in the development of postoperative hyphema. When clinically feasible, vitreous cavity tube insertion may reduce the risk of anterior chamber bleeding compared with anterior chamber or ciliary sulcus placement. In addition, careful early postoperative IOP management may help minimize hyphema risk. These findings may help surgeons better anticipate bleeding risk and optimize surgical strategies in patients undergoing BGI implantation.

In conclusion, postoperative hyphema following BGI surgery is primarily influenced by surgical factors, particularly tube insertion site, and early postoperative IOP. In eyes without NVG, vitreous cavity tube insertion is associated with a markedly lower hyphema risk compared to anterior chamber or ciliary sulcus placement, suggesting it may be a preferred approach when clinically feasible.

## Figures and Tables

**Table 1:** Patient characteristics.

Characteristics	Total (273)	Hyphema (+) (n = 77)	Hyphema (−) (n = 196)	*p* Value
Age, years	67.9 ± 13.2	68.9 ± 13.2	67.4 ± 13.2	0.29
Sex, n (%)				1.00
Male	173 (63.4)	49 (63.6)	124 (63.3)	
Female	100 (36.6)	28 (36.4)	72 (36.7)	
Diabetes mellitus, n (%)	114 (41.8)	29 (37.7)	85 (43.4)	0.42
Hypertension, n (%)	129 (47.3)	42 (54.5)	87 (44.4)	0.14
Antithrombotic therapy, n (%)	42 (15.4)	11 (14.3)	31 (15.8)	0.90
Glaucoma subtype, n (%)				0.22
Primary open-angle glaucoma	66 (24.2)	22 (28.5)	44 (22.5)	
Exfoliation glaucoma	69 (25.3)	20 (26.0)	49 (25.0)	
Primary angle-closure glaucoma	9 (3.3)	5 (6.5)	4 (2.0)	
Neovascular glaucoma	82 (30.0)	20 (26.0)	62 (31.6)	
Other secondary glaucoma	47 (17.2)	10 (13.0)	37 (18.9)	
Lens status, n (%)				0.30
Phakia	4 (1.5)	2 (2.6)	2 (1.0)	
Pseudophakia	224 (82.0)	67 (87.0)	157 (80.1)	
Aphakia	6 (2.2)	1 (1.3)	5 (2.6)	
Phakia combined with cataract surgery	39 (14.3)	7 (9.1)	32 (16.3)	
Previous intraocular surgeries, n	2.1 ± 1.1	2.2 ± 1.2	2.1 ± 1.0	0.59
Type of implant, n (%)				0.87
BG101-350	221 (81.0)	63 (81.8)	158 (80.6)	
BG102-350	52 (19.0)	14 (18.2)	38 (19.4)	
Endplate position, n (%)				0.48
Superotemporal	244 (89.4)	66 (85.7)	178 (90.8)	
Inferotemporal	9 (3.3)	3 (3.9)	6 (3.1)	
Superonasal	19 (6.9)	8 (10.4)	11 (5.6)	
Inferonasal	1 (0.4)	0 (0)	1 (0.5)	
Tube insertion site, n (%)				0.26
Anterior chamber	122 (44.7)	37 (48.0)	85 (43.4)	
Ciliary sulcus	79 (28.9)	25 (32.5)	54 (27.5)	
Vitreous cavity	72 (26.4)	15 (19.5)	57 (29.1)	
Preoperative IOP, mmHg	32.1 ± 9.6	31.1 ± 8.4	32.5 ± 10.1	0.53
Preoperative glaucoma medications, n	3.8 ± 1.2	3.8 ± 1.1	3.9 ± 1.3	0.42
Postoperative day 1 IOP, mmHg	20.8 ± 12.9	16.4 ± 13.0	22.6 ± 12.5	<0.01

IOP: intraocular pressure.

**Table 2:** Multivariable logistic regression analysis for postoperative hyphema after BGI surgery.

Variable	OR	95% CI	*p* Value
Age, per year	0.99	0.97–1.02	0.44
Antithrombotic therapy	0.67	0.28–1.49	0.33
Hypertension	1.73	0.95–3.16	0.071
Diabetes mellitus	0.81	0.35–1.83	0.62
Tube insertion site			0.074
Anterior chamber vs. Vitreous cavity	2.83	1.07–7.86	0.036
Ciliary sulcus vs. Vitreous cavity	2.88	1.10–7.82	0.031
Anterior chamber vs. Ciliary sulcus	0.98	0.50–1.93	0.96
Type of glaucoma (NVG/other)	1.35	0.52–3.55	0.54
Combined surgery (alone/combined cataract surgery)	1.92	0.76–5.36	0.17
Number of previous intraocular surgeries, per each	1.14	0.86–1.50	0.36
Preoperative IOP, per mmHg	0.99	0.96–1.02	0.50
Postoperative day 1 IOP, per mmHg	0.96	0.93–0.98	<0.01

OR: odds ratio, CI: confidence interval, IOP: intraocular pressure, NVG: Neovascular glaucoma.

**Table 3:** Multivariable logistic regression analysis for postoperative hyphema after BGI surgery (excluding NVG).

Variable	OR	95% CI	*p* Value
Age, per year	0.97	0.94–1.00	0.057
Antithrombotic therapy	0.68	0.21–1.95	0.48
Hypertension	1.82	0.88–3.85	0.11
Diabetes mellitus	0.78	0.30–1.88	0.58
Tube insertion site			<0.01
Anterior chamber vs. Vitreous cavity	15.63	2.48–312.59	<0.01
Ciliary sulcus vs. Vitreous cavity	22.02	3.29–452.13	<0.01
Anterior chamber vs. Ciliary sulcus	0.71	0.35–1.45	0.35
Combined surgery (alone/combined cataract surgery)	2.50	0.88–8.08	0.086
Number of previous intraocular surgeries, per each	0.93	0.62–1.36	0.69
Preoperative IOP, per mmHg	1.00	0.96–1.04	0.92
Postoperative day 1, IOP per mmHg	0.96	0.93–0.99	<0.01

OR: odds ratio, CI: confidence interval, IOP: intraocular pressure.

## Data Availability

All data generated or analyzed during this study are included in this published article.

## Abbreviations

The following abbreviations are used in this manuscript:

BGI	Baerveldt glaucoma implant
IOP	Intraocular pressure
NVG	Neovascular glaucoma
CI	Confidence interval
OR	Odds ratio
